# Identification of potential Mitogen-Activated Protein Kinase-related key genes and regulation networks in molecular subtypes of major depressive disorder

**DOI:** 10.3389/fpsyt.2022.1004945

**Published:** 2022-10-21

**Authors:** Youfang Chen, Feng Zhou, Weicheng Lu, Weian Zeng, Xudong Wang, Jingdun Xie

**Affiliations:** ^1^Department of Thoracic Oncology, Sun Yat-sen University Cancer Center, State Key Laboratory of Oncology in Southern China, Collaborative Innovation for Cancer Medicine, Guangzhou, Guangdong, China; ^2^Department of Neurology, First People’s Hospital of Foshan, Foshan, Guangdong, China; ^3^Department of Anesthesiology, Sun Yat-sen University Cancer Center, State Key Laboratory of Oncology in Southern China, Collaborative Innovation for Cancer Medicine, Guangzhou, Guangdong, China

**Keywords:** major depressive disorder, MAPK pathway, molecular subtypes, hub genes, regulation network

## Abstract

**Background:**

Major depressive disorder (MDD) is a heterogeneous and prevalent mental disorder associated with increased morbidity, disability, and mortality. However, its underlying mechanisms remain unclear.

**Materials and methods:**

All analyses were conducted based on integrated samples from the GEO database. Differential expression analysis, unsupervised consensus clustering analysis, enrichment analysis, and regulation network analysis were performed.

**Results:**

Mitogen-activated protein kinase (MAPK) signaling pathway was identified as an associated pathway in the development of MDD. From transcriptional signatures, we classified the MDD patients into two subgroups using unsupervised clustering and revealed 13 differential expression genes between subgroups, which indicates the probably relative complications. We further illustrated potential molecular mechanisms of MDD, including dysregulation in the neurotrophin signaling pathway, peptidyl-serine phosphorylation, and endocrine resistance. Moreover, we identified hub genes, including MAPK8, TP53, and HRAS in the maintenance of MDD. Furthermore, we demonstrated that the axis of miRNAs-TFs-HRAS/TP53/MAPK8 may play a critical role in MDD.

**Conclusion:**

Taken together, we demonstrated an overview of MAPK-related key genes in MDD, determined two molecular subtypes, and identified the key genes and core network that may contribute to the procession of MDD.

## Introduction

Major depressive disorder (MDD) is an increasingly multifactorial and devastating mental disorder that affects estimating 4.4% population of the world (1). It is usually prevalent throughout the lifespan and causes diverse somatic symptoms (2, 3). Recent work has demonstrated that MDD is associated with many other diseases, such as Alzheimer’s disease (4), Parkinson’s disease (5), and carcinoma (6). However, despite great advances in exploring the pathogenesis of MDD, the underlying mechanism remains largely elusive (7). Due to the lack of objective diagnostic tests, it is still hard to identify MDD patients and evaluate the status of MDD as early as possible (8). What’s worse, about one-third of patients treated with antidepressants do not reach symptomatic remission (9, 10). Although some candidate genes in MDD, like SLC6A4, have been identified, the procession is still unclear because of the genetic variants and environmental exposures (11). Hence, there is an urgent need to characterize specific and practical molecular signatures for accurate diagnosis and individualized treatment of MDD.

In the past few decades, many interpretations have been proposed to explore MDD (12, 13). It was noted that MDD is closely related to diverse brain region (14, 15), circadian genes (16), neuregulin signaling (17), insulin resistance (18), testosterone deficiency (19), neuroinflammation (20), and other metabolic pathways (21, 22). However, whether these pathways can be applied to identify MDD patients and evaluate their different statuses has not been fully elucidated.

In our study, we aimed to identify new significant and practical diagnoses and phased markers. We first identified a potential relative pathway by enrichment analyses based on the Differential Expression Genes (DEGs) from the GEO database.^1^ We next collected the most associated genes from GeneCards.^2^ Unsupervised consensus clustering further classified different subtypes of MDD patients. DEGs between subtypes were further identified. Enrichment analyses for the DEGs, including Gene Ontology (GO) function and Kyoto Encyclopedia of Genes and Genomes (KEGG) pathway analyses were performed. Furthermore, we recognized the hub genes and verified them by arrays expression from brain tissue. Finally, the protein-protein interaction (PPI) network and miRNAs-TFs-hub genes networks were constructed.

## Materials and methods

### Data preprocession and differential expression genes identification

**FIGURE 1:**
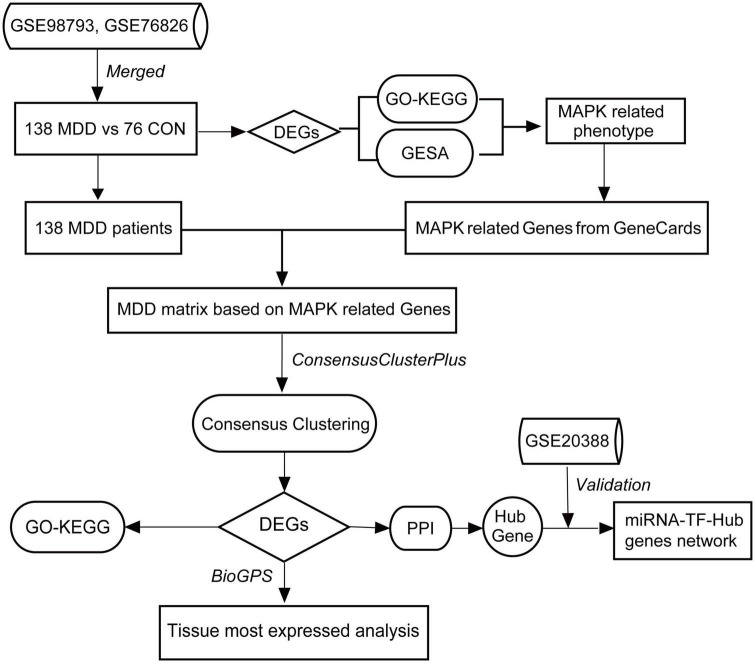
Flowchart of this study.

The datasets used in this research were downloaded from the GEO. The workflow of our study was shown in Figure 1. We selected two datasets: GSE98793 and GSE76826, including microarray RNA expression profiles and clinical data. The GSE98793 dataset includes 128 MDD patients and 64 healthy controls. The GSE76826 dataset includes 20 MDD patients and 12 healthy controls. But from GSE76826, we only chose the 12 healthy controls and 10 patients who were not in remission states [The depressive state was measured using the Structured Interview Guide for the Hamilton Depression (SIGH-D) rating scale; a remission state was defined as a stage in which a participant did not meet the diagnosis of a MINI major depressive episode for a period of 2 consecutive months and had a SIGH-D score of less than 8]. According to the annotation information on the GPL570 and GPL17077, the probes were respectively converted into corresponding gene symbols, and we next intersected common genes between two cohort data. According to the shared genes, two datasets were merged and normalized to remove the batch effect between arrays by the “SVA” R package (23). After screening, we finally gained 6,989 genes in a new dataset with 138 MDD patients and 76 healthy controls for analyses (Supplementary Table 1).

LIMMA package, meaning linear models for microarray data, was applied to distinguish DEGs between MDD patients and healthy controls in the integrated microarray expression matrix. Benjjammini-Hochberg’s method was used to control the false discovery rate (FDR). *P*-value < 0.05 and |log2 (fold change)| (|logFC|) > 0.3 were accepted as indicative of significant differences. Heatmap and volcano plots were constructed by the R packages “pheatmap” and “ggplot2.”

### Pathway enrichment and MAPK-related matrix construction

To further explore the potentially related pathway in MDD, we applied the “clusterProfiler” R package to perform gene set enrichment analysis (GSEA) (24). GO enrichment and KEGG pathway analyses. The “ggplot2” package visualized the results.

Based on the related mitogen-activated protein kinase (MAPK) pathway, we used the online resource Genecards to filter out the genes that may function in the MAPK pathway. The criteria for selection were listed as follows: category = “protein-coding,” score cut-off > 16. With the potential molecules in the MAPK pathway, we finally got a new correlative expression matrix through intersecting procession on the previously integrated matrix.

### Molecular subtypes identification and differential expression genes screening

Major depressive disorder patients samples were selected for further analyses. The R package “ConsensusClusterPlus” (24) (V1.54.0; parameters: reps = 50, pItem = 0.8, pFeature = 1, distance = “pearson”) was used for unsupervised consensus analysis. The consistent matrix (CM) plots, cumulative distribution function (CDF) index plot, Delta area plot, and tracking plot were constructed to determine our preferred *K* value. It was considered the best optimal when the CDF index was up to the approximate maximum. Based on the previous MAPK-related genes expression matrix, we validated the classification by principal components analysis (PCA) using the “clusterProfiler” R package.

The Limma in R was again used to identify the DEGs between clusters. *P*-value < 0.05 and | log FC| > 0.5 were considered statistically significant. The volcano plot of DEGs was present using the “ggplot2” R package, and the heatmap of DEGs was shown using the “pheatmap” R package.

### Tissue-most expressed gene analysis

To identify the most related tissues in different clusters of MDD patients, we used the online database BioGPS^3^ to search for the distribution of the DEGs. The most related tissues should be the top three expressed tissues for each gene, which can be identified as having a certain degree of specificity: (1) the most related tissues-expression level was more than the median, and (2) the fourth related tissue expression was less than one-third as high as the third level.

### Functional enrichment analyses of differential expression genes

In our study, functional enrichment analyses of DEGs containing GO terms and KEGG pathways were performed by “clusterProfiler” R package. Results with *P*-value < 0.05 were indicated as significant. The results of enrichment were further visualized by “ggplot2,” “ggnewscale,” and “enrichplot” packages in R.

### Construction and modular selection of protein-protein interaction networks

We applied STRING^4^ online tool to predict DEGs’ protein-protein interaction (PPI) network. A combined score ≥ 0.4 of PPI pairs was considered significant. The network of PPI was then sent to Cytoscape software (Version: 3.7.1) for visualization and subsequent analyses. Cytohubba application in Cytoscape was employed to identify the top 6 hub genes ranked by the MCC method (25). Moreover, the degree of each protein node was assessed by MCODE application in Cytoscape (26). Default criteria in MCODE plug-in were set as follows: Degree Cutoff = 2, Node Score Cutoff = 0.2, K-Core = 2, and Max. Depth = 100. We finally identified one module with 13 key genes, and their connection relation was visualized as a Sankey diagram plot by the “ggalluvial” R package.

### Expression validation of hub genes

To validate the expression of hub genes identified, we obtained another microarray dataset from GEO, GSE20388, an expression profile of a genetic animal model of depression. Eighteen samples from Flinders Depression Sensitive (FSL) cohort and 22 control samples from Flinders Depression Resistant (FRL) cohort were selected. All these samples were located in the Frontal Cortex. GEO2R, a functional tool in GEO,^5^ was used to verify the differential expression of hub genes. *P*-value of <0.05 was accepted as the significant threshold.

### Construction of miRNAs-TFs-hub genes network

To further illustrate the potential regulation network for the hub genes, we first applied three TF databases, including the ChEA3 online database,^6^ hTFtarget online database,^7^ and KnockTF online database^8^ to predict the transcription factors. A transcription factor would be included in the network only when it was indicated in more than two databases. We then intersected the TFs of the three hub genes and further predicted the upstream miRNAs for these common transcription factors using the ENCORI database (27). MiRNAs confirmed in the miRanda and TargetScan databases were selected. Finally, the network of miRNA-TF-hub genes was demonstrated using the Cytoscape software (Version: 3.7.1).

## Results

### Data preprocession and protein-protein interaction identification

**FIGURE 2:**
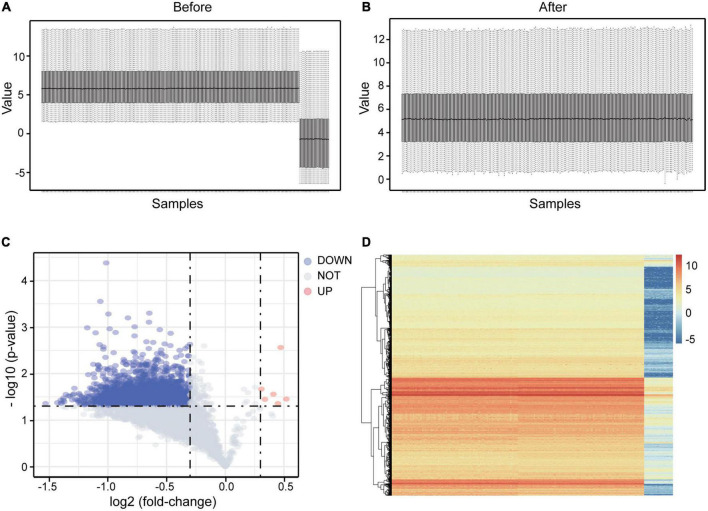
DEGs screening in integrated microarray of GSE98793 and GSE76826. **(A)** The boxplot showed an obvious batch effect before the data merged. **(B)** The boxplot showed the pleasant effect of data cleaning. **(C)** Volcano plot of the integrated exprSet. Data points in red represent up-regulated, and blue represent down-regulated genes. **(D)** Heatmap of DEGs identified in integrated microarray. Legend on the top right indicates the change of the genes.

From the GEO database, we obtained expression profiles from GSE98793 and GSE76826. With the preprocessing of the data mentioned above, we finally got a brand-new integrated microarray expression matrix with 6,989 genes and 214 samples (138 MDD patients and 76 healthy controls). Boxplot analysis indicated the effect of our data cleaning (Figures 2A,B).

Based on the limma package and previously set thresholds, a total of 3,122 DEGs for further enrichment were identified, including 3,116 down-regulated and 6 up-regulated DEGs. The volcano plot of DEGs was presented in Figure 2C, and the heatmap plot was shown in Figure 2D.

### Pathway enrichment and MAPK-related matrix construction

**FIGURE 3:**
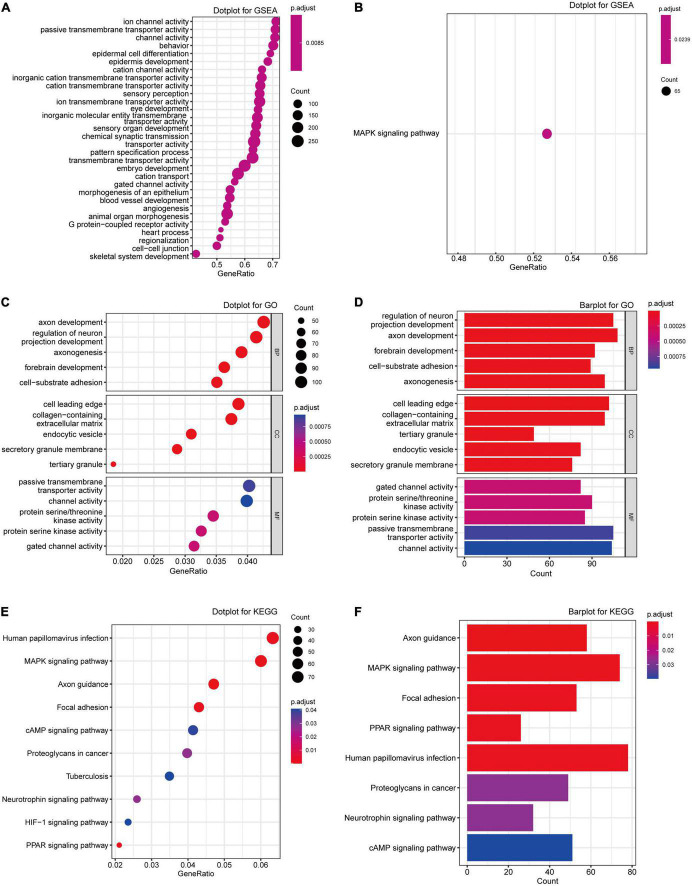
Enrichment analyses of the DEGs from exprset-merged. **(A,B)** GSEA analysis of the DEGs from exprset-merged. **(C,D)** GO enrichment analyses of the DEGs. **(E,F)** KEGG pathway enrichment analysis of the DEGs.

Gene set enrichment analysis was first performed to explore the most associated pathway. As Figure 3A shown, “ion channel activity,” “passive transmembrane transporter activity,” and “epidermis cell development” were the top GO terms enriched, while there was only MAPK signaling pathway enriched in Figure 3B. Further GO analysis also demonstrated that DEGs were mainly attributed to neuron regulation and different ion channel activities such as “axon development,” “passive transmembrane transporter activity,” “protein serine/threonine kinase activity,” and “gated channel activity” (Figures 3C,D; Supplementary Table 2). The pathway enrichment analysis indicated that the DEGs were enriched in the pathways such as “MAPK signaling pathway,” “Axon guidance,” “Focal adhesion,” and “Human papillomavirus infection” (Figures 3E,F; Supplementary Table 3). Considering all these results, we regarded the “MAPK pathway” as the most significantly enriched pathway in MDD.

Based on the MAPK signaling pathway, we searched associated genes in GeneCards. With the score cut-off > 16, we identified 30 genes for analysis (Supplementary Table 4). We then removed healthy control samples from the previous matrix. Subsequently, we conducted intersecting procession on a newly integrated matrix and obtained a MAPK-associated gene expression profile with 19 rows and 138 columns (Supplementary Table 5).

### Molecular subtypes identification and protein-protein interaction screening

**FIGURE 4:**
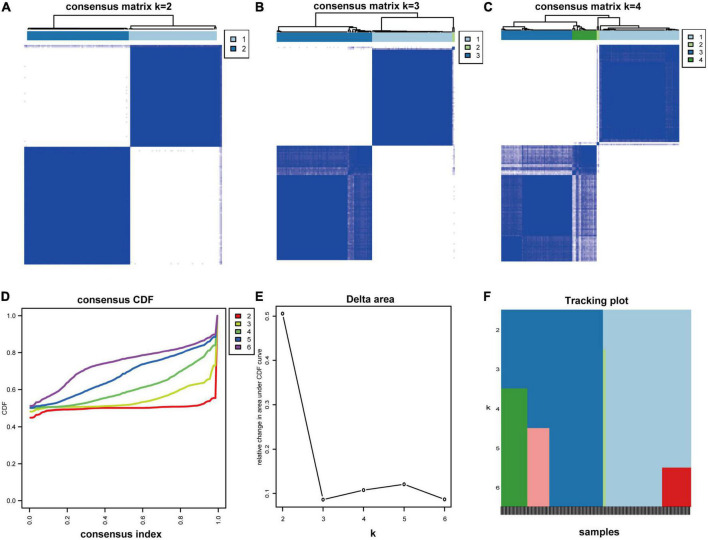
Consensus clustering analysis. **(A–C)** Consensus matrix for *k* = 2 to *k* = 4. **(D)** The CDF value of the consensus index. **(E)** Relative change in area under CDF curve for *k* = 2–6. **(F)** The tracking plot for *k* = 2 to *k* = 6.

To better characterize the MAPK-associated gene expression of 138 MDD samples, we applied the consensus clustering method to classify the patients. From the outcomes, the CM plot showed the maximum consistency at *k* = 2 (Figures 4A–C). When *K* = 2, the consensus CDF curve (Figure 4D), the Delta area plot (Figure 4E), tracking plot (Figure 4F) and item consensus plot (Supplementary Figure 1) consistently displayed the cluster stability. Thus, two subgroups named cluster 1 (64 MDD patients) and cluster 2 (74 MDD patients) were identified. Principal component analysis (PCA) also validated the differences between subgroups (Supplementary Figure 2).

**FIGURE 5:**
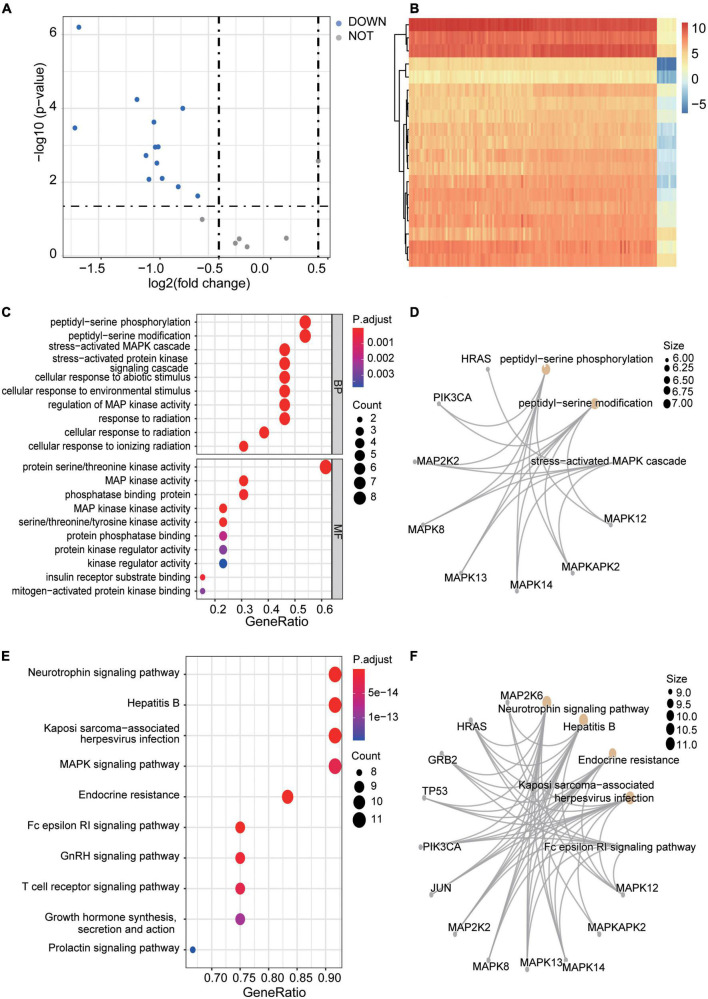
DEGs identification in subtypes and enrichment analyses. **(A)** A Volcano plot of the DEGs of exprSet after clustering. The blue nodes represent down-regulated DEGs. The dark nodes indicate Stable-genes. There is no up-regulated gene. **(B)** A heatmap of all DEGs of exprSet. Each column represents one sample, and each row represents one gene. The color changes from blue to red represents the changes from downregulation to upregulation for the expression. **(C,D)** The visualization of the results of GO enrichment analyses. **(E,F)** The visualization of the results of the KEGG pathway enrichment analysis.

Limma R package was then used to identify the DEGs. Statistical significance was defined as *P*-value < 0.05 and | logFC| > 0.5. Thirteen DEGs were finally determined to be significant, including 13 down-regulated genes but no up-regulated genes. The DEGs were further visualized by a volcano plot (Figure 5A) and a heatmap plot (Figure 5B).

### Tissue-most expressed analysis

To better know what symptoms the DEGs between different clusters may cause, we searched our DEGs in BioGPS. The most highly tissue-related expression system was the Immune system (46.2%, 6/13), while the circulatory system ranked second (38.5%, 5/13). Besides, neurological system (23.1%, 3/13) and respiratory system (23.1%, 3/13), endocrine system (15.3%, 2/13) and reproductive system (15.3%, 2/13) had similar levels of enrichment (Table 1).

**TABLE 1:** The most related tissues identified by BioGPS.

System	Tissue/Cell	Gene
Immune	Lymphoma_burkitts, CD33 + Myeloid, CD14 + Monocytes, CD19 + B cell, CD8 + Tcell	MAPK12, MAPK14, JUN, MAP2K2, MAP2K6, MAP2K6
Circulatory	Heart, blood, cardiac myocytes, atrioventricular node	MAPKAPK2, MAPK14, TP53, MAP2K6, SPRED1
Neurologic	Pineal, dorsal root ganglion, superior cervical ganglion, spinal cord, caudatenucleus	MAPKAPK2, MAPK8, HRAS
Respiratory	Brochial epithelial cells, CD105 + endothelial, lung	MAPK13, MAP2K2, HRAS
Endocrine	Pituitary, thyroid	MAPK8, SPRED1
Reproductive	Testis, testis Leydig cell	MAPK13, MAP2K2
Others	Skeletal muscle	MAPK12

BioGPS identified the related tissues according to the DEGs we filtered. They should have a certain degree of specificity: (1) the most related tissues expression level was more than the median, and (2) the fourth related tissue expression was less than one-third as high as the third level.

### Gene ontology and KEGG pathway enrichment

Functional enrichment analyses of 13 DEGs were conducted. Results showed that DEGs were mainly enriched in “peptidyl-serine phosphorylation,” “peptidyl-serine modification,” “stress-activated MAPK cascade,” “stress-activated protein kinase signaling cascade,” “cellular response to abiotic stimulus,” and “protein serine/threonine kinase activity” (Figures 5C,D). *P* < 0.05 and enriched genes count > 5 were further set as screening criteria, the top 6 GO terms were additionally present in Supplementary Table 6. Meanwhile, 13 DEGs were also found enriched in some pathways like “Neurotrophin signaling pathway,” “Hepatitis B infection,” “MAPK signaling pathway,” “Endocrine resistance,” and “Fc epsilon RI signaling pathway” et al. (Figures 5E,F). Further, the top 6 enriched KEGG pathways with enriched genes count > 8 were further screened and shown in Supplementary Table 7.

### Construction of the protein-protein interaction network and module identification

**FIGURE 6:**
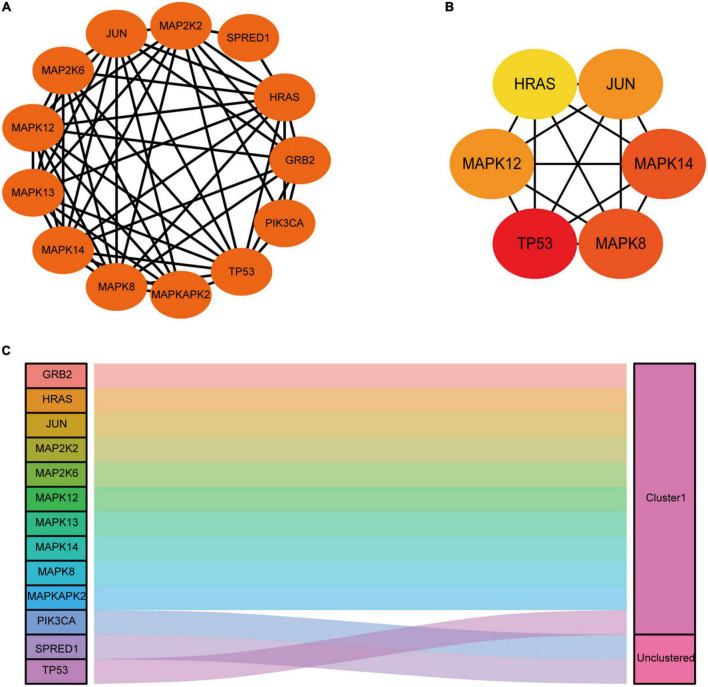
Construction of functional networks and identification of candidate genes and module analysis. **(A)** Functional protein-protein interaction (PPI) network analysis of the 13 differentially expressed genes (DEGs). **(B)** Subnetwork of top six hub genes from the PPI network. Node color reflects the degree of connectivity (Red color represents a higher degree, and yellow color represents a lower degree). **(C)** One module was identified by ggalluvial in R and was visualized by ggplot.

From the STRING database (see text footnote 4), we identified a PPI network consisting of 13 nodes meaning DEGs and 52 edges to display their relationship. PPI network was further imported into Cytoscape for visualization (Figure 6A) and subsequent analysis. Cytohubba application in Cytoscape was applied to identify the top 6 hub genes by the MCC method (Figure 6B). TP53, MAPK12, MAPK14, JUN, MAPK8, and HRAS were screened with the highest connectivity. Then, we further identified a notably significant cluster of 11 genes, including GRB2, HRAS, JUN, MAP2K2, MAP2K6, MAPK12, MAPK13, MAPK14, MAPK8, MAPKAPK2, and TP53. The result of the module was presented as a Sankey diagram plot (Figure 6C).

### Expression validation of hub genes

We verified the expression of hub genes using the GSE20388 dataset derived from the depression animal brain tissue. We found that 4 out of the 6 hub genes show the same significant differential expression. But only 3 hub genes exhibited the same expression trend, including TP53, MAPK8, and HRAS (Table 2). Therefore, we chose TP53, MAPK8, and HRAS for the following analyses.

**TABLE 2:** DEGs identification from different clusters of MDD.

Gene	Log2FC	AveExpr	t	*P*-value	Adj. *P*-val	B	Change	Change in GSE20388
MAPK12	−3.0576	3.40516	−5.1085	9.37E-07	3.56E-06	5.16614	DOWN	NOT
MAPKAPK2	−2.6175	9.74616	−6.2793	3.23E-09	6.14E-08	10.6347	DOWN	DOWN
MAPK8	−2.1538	6.05629	−4.3216	2.75E-05	5.22E-05	1.94359	DOWN	DOWN
MAPK13	−2.0476	6.53489	−4.6987	5.71E-06	1.36E-05	3.43763	DOWN	–
MAPK14	−1.9784	7.51392	−5.4315	2.11E-07	2.00E-06	6.59983	DOWN	NOT
JUN	−1.8926	5.36798	−4.574	9.70E-06	2.05E-05	2.93307	DOWN	UP
MAP2K2	−1.8222	8.4757	−4.8141	3.47E-06	9.41E-06	3.91366	DOWN	DOWN
TP53	−1.7985	5.22376	−4.2978	3.03E-05	5.23E-05	1.85267	DOWN	DOWN
MAP2K6	−1.7603	6.41526	−5.1478	7.84E-07	3.56E-06	5.33699	DOWN	NOT
PIK3CA	−1.7517	6.78338	−4.8147	3.46E-06	9.41E-06	3.91627	DOWN	DOWN
SPRED1	−1.6162	2.68101	−4.1456	5.55E-05	8.79E-05	1.27957	DOWN	–
BRAF	−1.508	5.89715	−3.5013	0.0006	0.00082	−0.9571	DOWN	–
HRAS	−1.2981	5.0562	−3.9696	0.00011	0.00016	0.63786	DOWN	DOWN

DEGs between different clusters were screened with *P*-value < 0.05 and | log FC| ≥ 0.5. All of them were verified by GSE20388.

### MiRNA-TFs-hub genes network construction

**FIGURE 7:**
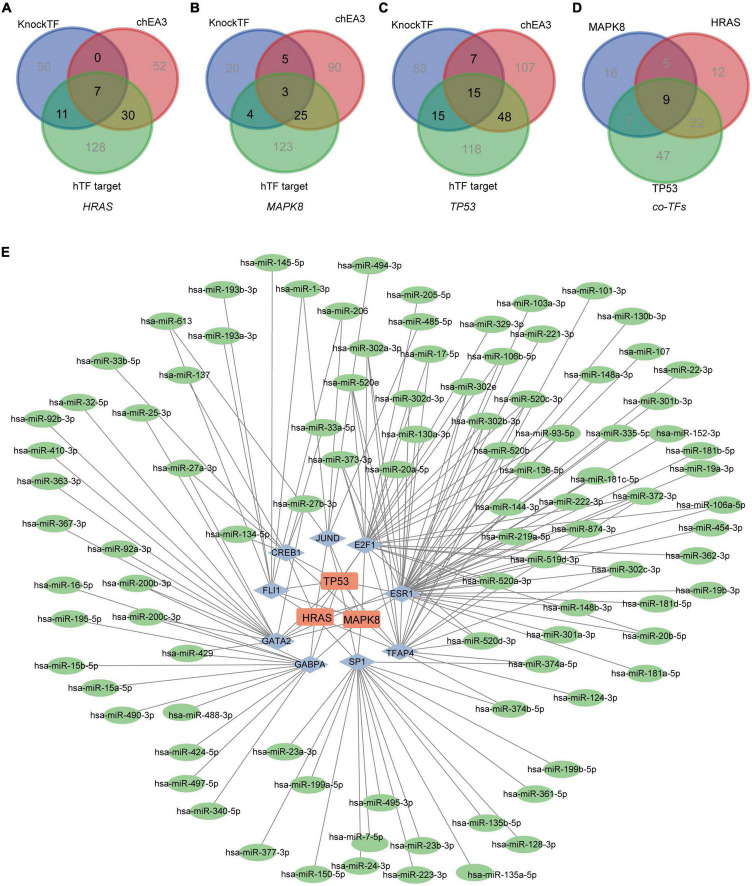
Network of miRNAs-TFs-hub genes. **(A–C)** The predicted TFs for hub genes based on the KnockTF, chEA3, and hTFtarget databases, respectively. **(D)** The common TFs for HRAS, MAPK8 and TP53. **(E)** The miRNAs-TFs-hub genes regulation network in MDD. (The Square nodes represent the hub genes, and diamond nodes represent the TFs. The circle nodes represent the miRNAs.)

Based on three TF databases, we predicted different TFs and took the results’ intersection for the above hub genes, respectively. Forty-eight TFs for HRAS, 37 TFs for MAPK8, and 85 TFs for TP53 were identified (Figures 7A–C). By taking a further intersection, 9 co-TFs including GATA2, FLI1, CREB1, JUND, E2F1, ESR1, TRAP4, SP1, and GABPA were selected (Figure 7D). Based on the ENCORI database, multiple upstream miRNAs for co-TFs were further screened. The Cytoscape software demonstrated the regulation network of miRNAs-TFs-hub genes (Figure 7E). Taken together, we supposed that the axis of miRNAs-TFs-HRAS/TP53/MAPK8 may play a potentially important role in MDD.

## Discussion

Major depressive disorder is a debilitating disorder closely associated with AD and other diseases. Most recent studies have revealed pathways and biomarkers attributable to it. Nevertheless, the exact mechanisms of MDD remain widely unclear. In our study, we identified 3,116 down-regulated and 6 up-regulated genes between MDD patients and healthy controls. By enrichment analysis, we demonstrated that the MAPK signaling pathway, passive transmembrane transporter activity and ion channel activity might play an essential role in the development of MDD. The terms of passive transmembrane transporter activity and ion channel activity can regulate energy metabolism, thus modulating various clinical symptoms. And the MAPK pathway has also been reported to be correlated with MDD (28). However, it still lacks more detailed studies of the correlation.

Based on the 30 MAPK-associated genes from Genecards, we performed unsupervised consensus clustering on the MAPK-related genes expression matrix. Two subgroups named cluster 1 (64 MDD patients) and cluster 2 (74 MDD patients) were identified. We then screened 13 down-regulated genes between different clusters. The DEGs were mainly distributed in tissues of the circulatory system, immune system, and neurologic system, consistent with the fact that MDD patients usually harbor coronary artery disease (29). In our study, the most typical tissues were cardiomyocytes, lymphoma Burkitt, CD33+ myeloid, pineal, and Dorsal Root Ganglion, which might imply the probable reasons for clinical manifestations in MDD, such as repeated infection, circadian rhythm disorder and feeling pain (30, 31).

MF analysis in GO annotation for DEGs demonstrated that 13 down-regulated genes were significantly enriched in protein serine/threonine kinase activity. And the kinase-associated pathway was also validated by KEGG analysis. Meanwhile, the pathway of MAPK cascade, stress-activated protein kinase signaling cascade and cellular response to abiotic stimulus were also identified. As previously described, the results confirmed that MDD is closely associated with stress and external stimulation (32). Additionally, the correlation among the DEGs was visualized by the PPI network. And a module constructed by MCODE was demonstrated. Besides, 3 hub genes including MAPK8, TP53, and HRAS were further identified and verified.

MAPK8 is a typical member of the MAP kinase family. It was confirmed that the MAPK pathway is probably closely associated with MDD. TP53, also known as the cancer suppressor gene, is closely correlated with transcriptional activation, DNA binding and oligomerization domains. It has been implicated that TP53 can induce cell cycle arrest, senescence, and changes in metabolism (33), especially when responding to complicated cellular stresses. TP53 mutation is also an independent risk factor for immune escape (34). Though some reports noted that the mechanisms involved in cell survival and death regulation based on TP53 might be interested in the pathophysiology of MDD (35), there is no more indication about TP53 and MDD. To some extent, we inspired a new light on the association between the traditional molecule and MDD. HRAS, a gene belonging to the Ras oncogene family, was also identified in our study. Evidence has suggested that HRAS is closely associated with Beta-Adrenergic signaling, which controls cell migration and TP53-dependent cell survival (36). It has also been revealed that the mutation of HRAS is closely associated with TP53 in the immune signature (37, 38). All these DEGs were supposed to participate in different metabolism pathways like phosphorylation, which has been reported as an essential alteration in MDD (39, 40). Accordingly, we can early discriminate MDD through the expression of hub genes, preventing further exacerbation of the disease. And perhaps our results contribute to explaining why MDD patients are clinically prone to tumors and other immune system-related diseases.

We also constructed miRNAs-TFs-hub genes regulation network in MDD. The common transcription factors were GATA2, FLI1, CREB1, JUND, E2F1, ESR1, TRAP4, SP1, and GABPA. It has been reported that GATA2, CREB1, and E2F1 were crucial in the maintenance of MDD (41–43). And ESR1, an estrogen receptor, was also found to be important in depression (44). Meanwhile, the role of SP1 in MDD has also been identified (45). Although studies about the other TFs in MDD are limited, they are still potential targets for MDD that worth exploring in the future. Besides, some research has reported that miRNA may act as potential biomarkers for psychiatric and neurodegenerative disorders (46, 47). For example, the miR-29 family, miR-34a-5p, and miR-132-3p were discussed as common dysregulated circulating miRNA in CNS disorders (48). And from Zheng K’s work, miR-135a-5p was demonstrated to be a synaptic-related regulator (49). Our constructing network also demonstrated potential regulation miRNAs, including the hsa-miR-134-5p, hsa-miR-27b-3p, hsa-miR-373-3p, and hsa-520a-3p, et al.

There are still several issues to be addressed. First, there were shortcomings in our integrated expression matrix, for the platforms were different (GPL570 for GSE98793 and GSE17077 for GSE76826). The batch effect cannot be eliminated entirely though using the “SVA” R package. Second, both GSE98793 and GSE76826 lack sufficient clinical information, causing the absence of more clinical analyses. Furthermore, the expressions of the hub genes and their roles in MDD need to be further explored and assessed *in vivo* and *in vitro*.

## Conclusion

Briefly, we demonstrated an overview of MAPK-related key genes in MDD. Two distinct molecular subtypes of MDD were identified, which exhibit differential expression of GRB2, HRAS, JUN, MAP2K2, MAP2K6, MAPK12, MAPK13, MAPK14, MAPK8, MAPKAPK2, and TP53. The DEGs indicated a significant correlation between MDD and clinical somatic symptoms like infection, circadian rhythm disorder and feeling pain. A new module consisting of GRB2, HRAS, JUN, MAP2K2, MAP2K6, MAPK12, MAPK13, MAPK14, MAPK8, MAPKAPK2, and TP53 was further identified, better illustrating the potential regulation of MDD. Moreover, we distinguished TP53, MAPK8 and HRAS as hub genes and demonstrated an axis of miRNAs-TFs-HRAS/TP53/MAPK8. These findings highlight the role of the MAPK pathway in MDD and provide insights into diagnosis and therapy in the future.

## Data availability statement

Publicly available datasets were analyzed in this study. This data can be found here: NCBI: GSE98793, GSE76826, and GSE20388.

## Author contributions

JX and XW designed the study. YC, FZ, and WL collated the data, carried out data analyses, and produced the initial draft of the manuscript. WZ helped perform the analysis with constructive discussions. All authors have read and approved the final submitted manuscript.

## Footnotes

1
http://www.ncbi.nlm.nih.gov/geo

2
https://www.genecards.org

3
http://biogps.org

4
http://www.string-db.org/

5
https://www.ncbi.nlm.nih.gov/geo/geo2r

6
https://amp.pharm.mssm.edu/ChEA3

7
http://bioinfo.life.hust.edu.cn/hTFtarget/

8
http://www.licpathway.net/KnockTF/

## Supplementary material

Supplementary Figure 1 Item consensus plot displayed the cluster stability at *k* = 2. **(A–C)** Item consensus plot showed that when *k* = 2, the cluster reached its stability.

Supplementary Figure 2 PCA visualization showed a significant difference between subgroups.
