# Supplementary figures and images for: Identification of potential Mitogen-Activated Protein Kinase-related key genes and regulation networks in molecular subtypes of major depressive disorder
Source: Front Psychiatry. 2022 Oct 21;13:1004945. doi: 10.3389/fpsyt.2022.1004945 (PMC9634261; doi:10.3389/fpsyt.2022.1004945)

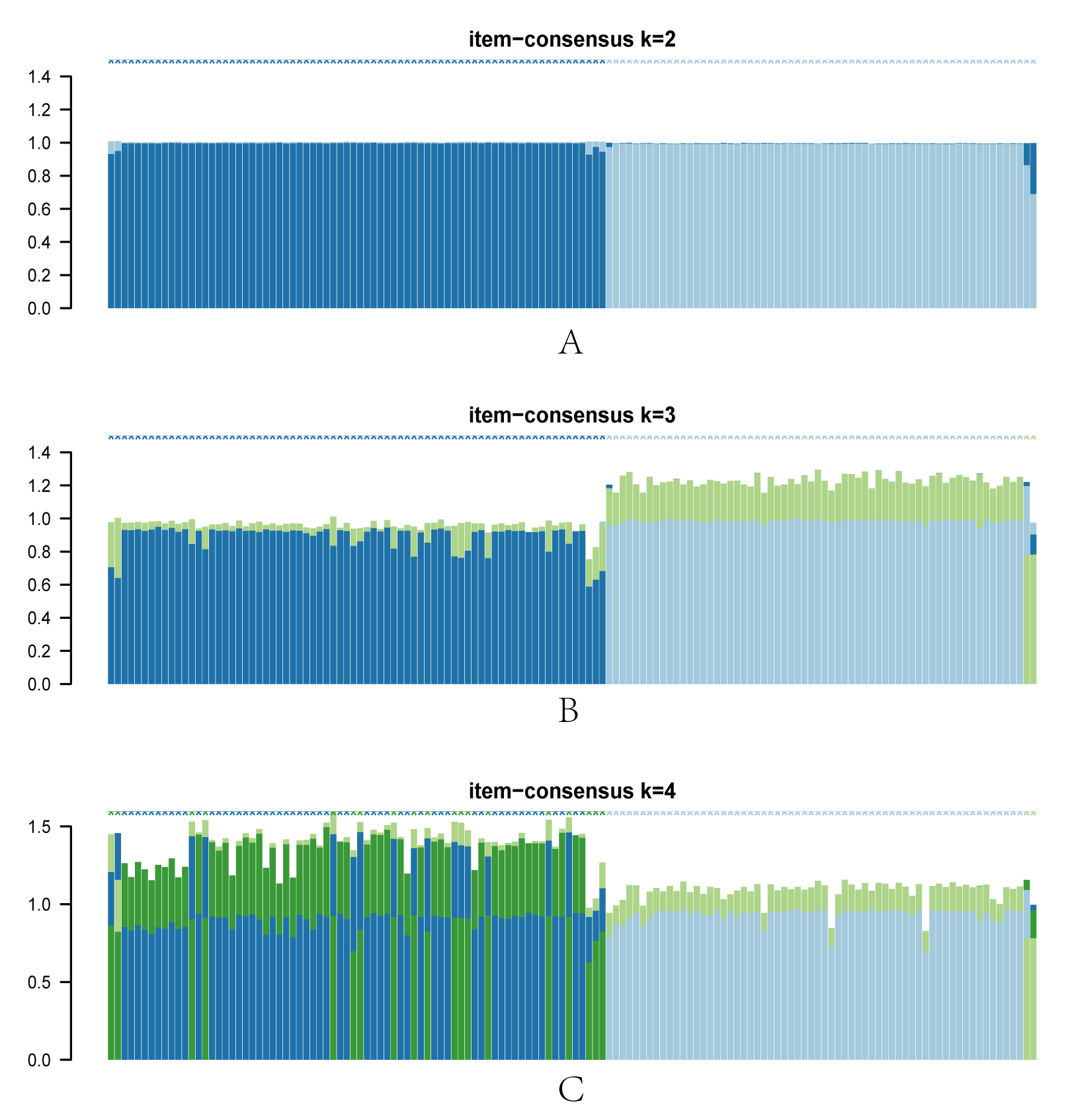

Supplement: Supplementary Figure 1 — Item consensus plot displayed the cluster stability at k = 2. (A–C) Item consensus plot showed that when k = 2, the cluster reached its stability. [file Image_1.TIF]

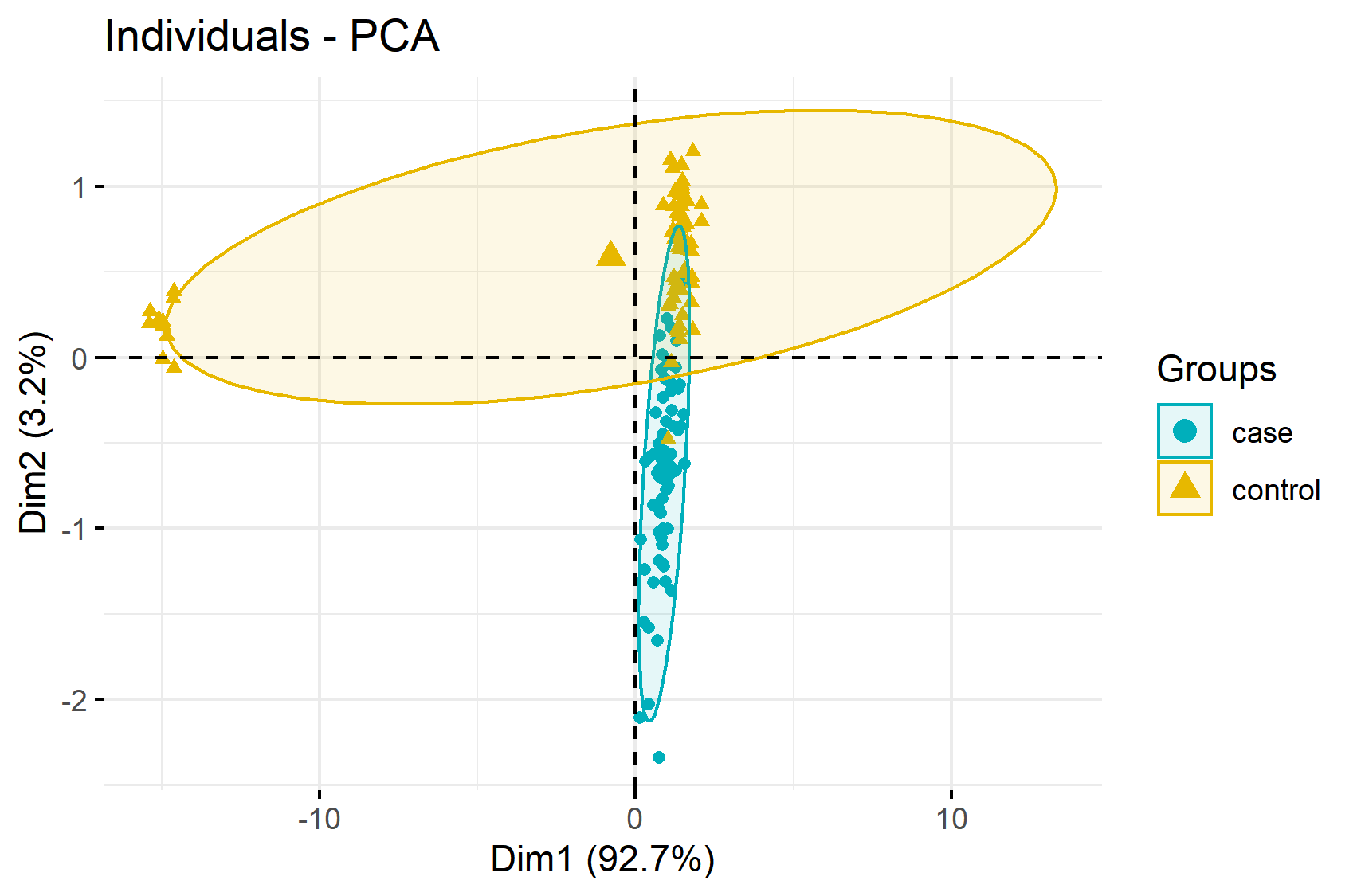

Supplement: Supplementary Figure 2 — PCA visualization showed a significant difference between subgroups. [file Image_2.TIF]
